# Percutaneous CAPD catheter insertion by a nephrologist versus surgical placement: A comparative study

**DOI:** 10.4103/0971-4065.41280

**Published:** 2008-01

**Authors:** K. Sampathkumar, A. R. Mahaldar, Y. S. Sooraj, M. Ramkrishnan, Ajeshkumar, R. Ravichandran

**Affiliations:** Department of Nephrology, Meenakshi Mission Hospital and Research Centre, Lake area, Madurai - 625 107, India; 1 Department of Urology, Meenakshi Mission Hospital and Research Centre, Lake area, Madurai - 625 107, India

**Keywords:** Continuous ambulatory peritoneal dialysis, interventional nephrologists, percutaneous insertion, peritoneal dialysis catheter

## Abstract

Peritoneal dialysis catheter (PDC) for continuous ambulatory peritoneal dialysis is inserted into the abdominal cavity either by a surgeon, interventional radiologist or nephrologist. Various innovations have been made in the methodology adopted in the placement of the PDC. We compared the percutaneous approach for PDC insertion with the open surgical technique. From January 2006 to May 2007, 25 of the 46 catheters were successfully inserted using the percutaneous Seldinger technique. The incision size (2.6 ± 0.7 vs 7.3 ± 0.6 cm) and the length of hospital stay (11.9 ± 5.9 vs 17.3 ± 6.8 d) were considerably less in the percutaneously placed group compared to the surgically placed group. Early initiation of exchanges and reduction in the expenses were other important advantages of this method.

## Introduction

The care of chronic kidney patients frequently involves diagnostic and interventional radiological procedures such as diagnostic renal ultrasonography, ultrasound-guided kidney biopsies, placement of tunneled hemodialysis or peritoneal catheters, sonographic and radiological investigation of vascular access dysfunction. Presently, most of these procedures are performed by radiologists, vascular surgeons and surgeons. This fragmentation does not optimize medical care, and is inconvenient to the patient. This has led many nephrologists to introduce a new paradigm in the management of kidney patients, often referred as interventional nephrology (IN). This new class of nephrologists has acquired diagnostic and interventional skills for procedures usually performed by other specialists with an added clinical perspective.^1^

Surgical implantation of the peritoneal dialysis catheter (PDC) requires a laparotomy procedure or at least a laparoscopy for minimal access. These operations entail a significant morbidity in the form of postoperative pain and immobility besides straining the financial resources. Percutaneous PDC placement can be performed by the nephrologist to provide a quick, safe and reliable peritoneal access.

## Materials and Methods

We retrospectively studied 46 consecutive patients in whom CAPD was initiated at our center. We collected the data regarding the demography, etiology, procedure, complications and fiscal considerations. Patients with previous abdominal surgery or severe liver disease were not included. Most of the surgically placed catheters were placed between January and August 2006; most of the percutaneously placed catheters were placed from September 2006 onwards by the nephrologist. Patient selection for percutaneous or surgical placement was not randomized. We used double-cuffed Tenckhoff catheters with straight tips (Quinton Instrument Company, Seattle, WA, USA).

Surgical insertion (placement by dissection) was performed using the paramedian or lateral approach.^2^ The ratio of specialist/nonspecialist was identical in the surgery and nephrology teams, and all procedures were performed in the presence of at least one attending physician or specialist.

### Percutaneous catheter insertion by a nephrologist

We performed blind placement based on the Seldinger technique^3^ using local anesthesia.

Antibiotic prophylaxis was administered with intravenous vancomycin (1 g) administered 2 h prior to the procedure. Patients received intravenous fentanyl (1 μg/kg) or propofol (1 mg/kg) and local anesthesia (2% lignocaine). A horizontal paramedian incision, 2-3-cm long, was made; followed by blunt dissection of subcutaneous tissue until the fascia of the rectus muscle. The peritoneum was punctured using a 16-gauge needle from the Quinton-catheter placement kit. The position of the guidewire was confirmed with fluoroscopy using image intensifier. A peel-away sheath and introducer were inserted over the guidewire. The introducer was removed along with the guidewire leaving the peel-away sheath in situ. The PDC was advanced through the peel-away sheath and directed caudally toward the left iliac fossa thus splitting the peel-away sheath. The position of the PDC was reconfirmed with fluoroscopy to ensure its positioning in the pelvis. The inner cuff of PDC was secured by a suture on the fascia of the rectus muscle. An 8-12-cm subcutaneous tunnel for the PDC was fashioned by using a stylet. The proximal end of the PDC was pulled through the exit site and positioned in a manner that the inner cuff was located at the peritoneal entry at the fascia of the rectus muscle, and the second cuff was 2 cm away from the exit site. The original incision was then closed and the PDC was flushed with 2 L of heparinized 2.5% dialysis solution to confirm catheter patency and examine intra-abdominal bleeding. The line was then capped-off unless there was significant blood staining of the effluent. If the latter occurred, hourly cycles were continued until the drained dialysate was clear.

CAPD was initiated 3-7 d after PDC placement. Patient training was performed during this period. Low volume supine exchanges (up to 250 mL) were periodically performed during the training, and patients were instructed to avoid constipation. The results are summarized in Table 1.

**Table 1:** Comparison of percutaneous vs surgical placement of catheter

	Percutaneous	Surgical	*P* value
Number of cases	25	21	-
Follow-up (days)	238.35 ± 96.23	408.20 ± 172.84	<0.001*
Age (years)	53 ± 10.7	56.8 ± 13.637	0.511
Size of incision (cm)	2.6 ± 0.707	7.3 ± 0.65	<0.001*
Break in period (days)	4.6 ± 2.44	6.31 ± 2.688	0.119
Hospital stay (days)	11.96 ± 5.711	17.35 ± 6.853	0.008*
Leakage	3 (12)	0	-
Bowel injury	1 (4)	0	-
Conversion to surgical placement	2 (8)	0	-
Average expenditure (INR)	19000 ± 3687	35500 ± 11000	0.000*

* Numbers in parentheses are percentages

## Discussion

The present report suggests that percutaneous insertion of PDCs is a dependable peritoneal access technique, and is compared favorably with surgical techniques in terms of catheter-related mechanical complications. This strategy has the added advantages of early initiation of exchanges without a break-in period and reduced expenditure.

Although percutaneous insertion has been reported to be safe, previous reports have showed a high incidence of leakages and early mechanical complications and the potential risk of bowel perforation since this technique is a ‘blind’ procedure without direct visualization of the peritoneum.^4^–^6^

Percutaneous bedside placement of PDCs by nephrologists has been demonstrated to be a safe and reliable.^7^ Several innovations have been described recently. Zaman F *et al.* adopted a percutaneous approach with fluoroscopic guidance for PDC insertion and demonstrated it to be convenient and safe and showing good patency and infection rate results.^8^ This technique involves the instillation of radiocontrast dye into the peritoneal cavity. A new laparoscopic technique using an extraperitoneal approach with omentopexy for PDC placement has proved to be extremely useful for preventing catheter malfunction caused by catheter tip migration, pericatheter leakage, omental wrapping and periodic catheter movement that causes abdominal pain in CAPD.^9^ The use of laparoscopy mandates general anesthesia and is associated with increased expenditure.

With experience, the rates of pericatheter leakage and other catheter-related complications have been shown to be relatively low in CAPD patients using percutaneous catheter placement method without a break-in procedure.^10^ This procedure is comparatively simple and less invasive than other catheter placement methods and permits immediate initiation of PD after catheter insertion, without a break-in procedure. Short hospital stay entails less expenditure and earlier institution of renal replacement therapy for severely uremic patients.

Based on the literature survey, we placed percutaneous PDCs using local anesthesia supplemented with intravenous (IV) analgesia with fluoroscopic guidance. The break-in period was shorter than the conventional two-week period.

In our study, early leakage was observed in 12% of the percutaneously inserted catheters. All these were resolved after a waiting period of one week before resuming the cycles. Among the percutaneously placed PDCs, early leakage varied from 2.6% to 22%.^11^,^12^ Moreiras *et al.* reported that 15.3% of their mechanical complications were related to the insertion and 6% to early leakage.^11^ In the study conducted by Smith *et al.*, the most common early complication was leakage (13%) and bleeding that rapidly resolved with repeated exchanges (2/31 catheters).^13^ Allon *et al.* reported that 19 of the 154 percutaneously placed catheters demonstrated early complications, and early leakage was observed in 2.6%.^12^ Swartz *et al.* reported early leakage as high as 21.6%.^14^ Reports regarding leakage from surgical studies vary between 0.9% and 8.6%.^4^,^5^ A low incidence of leakage in our percutaneous group was probably due to the lateral placement of the inner cuff and appropriate fixation in the rectus muscle using a paramedian incision, as described in previous reports.^6^ In addition, we avoided using any force during catheter insertion.

The major complication of the percutaneous placement as a ‘blind’ technique is the risk of inadvertent puncture of the abdominal viscera. However, very low (0-1.3%) frequency of perforations reported in previous percutaneous studies argues against the magnitude of this complication.^3^,^11^,^13^,^15^ In our study, we encountered one episode of minor bowel perforation, which resolved without intervention. Although mechanical complications remain the major cause of catheter removal in both surgical and percutaneous techniques, no significant difference was observed in the rate of mechanical complications related to catheter insertion between the two groups.

Catheter-related malfunction causing drainage failure may arise following the obstruction of the catheter or migration of the catheter tip from the pelvis into the upper abdomen. The incidence of catheter-related malfunction varies from 0.9% to 17% for surgical technique^4^ and from 4% to 21% for percutaneous technique.^3^,^11^–^13^,^15^ Although it has been argued that surgical catheter placement is preferable to percutaneous placement because of the direct visualization during positioning,^16^ several studies have shown that there is no advantage of surgical placement with regard to catheter-related malfunction.^15^,^17^ Our data supports this view, with only one preperitoneal placement in the percutaneous implantation group.

The incision size was 2.6 ± 0.707 cm in the percutaneous group (P), while that in the surgical group (S) was significantly larger at 7.3 ± 0.65 cm. A large incision size also resulted in a longer hospital stay of approximately 17.35 ± 6.85 d in surgical group, while the percutaneous group patients were admitted for 11.96 ± 5.711 d. The cost-effective analysis reveals a significant advantage with the percutaneous insertion of PDC. The average expense to the patients in group P was INR 19000 ± 3687, while that to the patients in group S, significantly higher at INR 35500 ± 11000. The convenience in performing the procedure, early initiation of dialysis and saving expenses are ideal for patients in developing countries such as India since patients come to nephrologists' notice quite late and have limited financial resources.

## Conclusions

The present study regarding percutaneously placed PDCs clearly demonstrates that in the hands of experienced nephrologists and CAPD nurses, and with adequate education and training regarding CAPD patients, the percutaneous technique can be used to provide a reliable, safe and cost-effective method for the placement of PDCs thus ensuring early initiation of renal replacement therapy.
